# Use of Digital Methods to Optimize Visualization during Surgical Gonioscopy

**DOI:** 10.3390/jcm12082794

**Published:** 2023-04-10

**Authors:** Otman Sandali, Mohamed El Sanharawi, Rachid Tahiri Joutei Hassani, Ashraf Armia Balamoun, Cedric Duliere, Sidi Mohammed Ezzouhairi, Ahmed B. Sallam, Vincent Borderie

**Affiliations:** 1 Centre Hospitalier National d’Ophtalmologie des XV-XX, 28 Rue de Charenton, 75571 Paris, France; 2 Service de Chirurgie Ambulatoire, Hôpital Guillaume-de-Varye, 18230 Bourges, France; 3 Service d’Ophtalmologie, Centre Hospitalier de Châteaudun, 28200 Châteaudun, France; 4 Service de Chirurgie Ambulatoire, Centre Hospitalier de Granville, 50400 Granville, France; 5 Watany Eye Hospital, Cairo 11775, Egypt; 6 Watany Research and Development Centre, Cairo 11775, Egypt; 7 Ashraf Armia Eye Clinic, Giza 12655, Egypt; 8 Hôpital Lariboisiere, 75010 Paris, France; 9 Glaucoma Center, Mohammedia 28800, Morocco; 10 Department of Ophthalmology, Harvey and Bernice Jones Eye Institute, University of Arkansas for Medical Sciences, Little Rock, AR 72205, USA

**Keywords:** surgical gonioscopy, heads-up three-dimensional (3D) digital visualization system, color filter, color temperature, color saturation, contrast, visualization, stent implantation, trabecular meshwork, Schlemm’s canal

## Abstract

**Purpose:** The aim of this study was to evaluate the efficacy of digital visualization for enhancing the visualization of iridocorneal structures during surgical gonioscopy. **Methods:** This was a prospective, single-center study on a series of 26 cases of trabecular stent implantation performed by the same surgeon. Images were recorded during surgical gonioscopy, and before stent implantation, with standard colors and with the optimization of various settings, principally color saturation and temperature and the use of the cyan color filter. Subjective analyses were performed by two glaucoma surgeons, and objective contrast measurements were made on iridocorneal structure images. **Results:** The surgeons evaluating the images considered the optimized digital settings to produce enhanced tissue visibility for both trabecular meshwork pigmentation and Schlemm’s canal in more than 65% of cases. The mean difference in the standard deviation of the pixel intensity values was 37.87 (±4.61) for the optimized filter images and 32.37 (±3.51) for the standard-color images (*p* < 0.001). The use of a cyan filter provided a good level of contrast for the visualization of trabecular meshwork pigmentation. Increasing the color temperature highlighted the red appearance of Schlemm’s canal. **Conclusions:** We report here the utility of optimized digital settings including the cyan filter and a warmer color for enhancing the visualization of iridocorneal structures during surgical gonioscopy. These settings could be used in surgical practice to enhance the visualization of the trabecular meshwork and Schlemm’s canal during minimally invasive glaucoma surgery.

## 1. Introduction

Minimally invasive glaucoma surgery (MIGS) has emerged in recent years as a new therapeutic option for glaucoma surgery. MIGS is less invasive than conventional glaucoma surgery, making it possible to achieve minimal tissue disruption more safely and with faster recovery [1].

Schlemm’s canal devices are inserted using an ab interno method, with the assistance of a gonioscopic lens, to increase aqueous humor outflow through the conventional pathway [2,3]. Good visualization of the Schlemm’s canal target is, therefore, essential to ensure the success of MIGS for enhancing aqueous humor drainage through the collector channels.

The more pigmented areas of the TM have been reported to be located in close proximity to collector channels and can serve as a visual guide during MIGS [4]. However, in patients with little or no pigmentation, the iridocorneal structures, including Schlemm’s canal in particular, are more difficult to identify, potentially jeopardizing the correct implantation of MIGS devices.

A recently developed three-dimensional (3D) heads-up system for use in both cataract and vitreoretinal surgery can markedly improve visualization quality during these operations. The advantages of this system over conventional microscopes include its ocular-free design, larger depth of field, and digitally enhanced imaging, resulting in high-quality visualization [5,6,7,8].

In a prospective series, we recently demonstrated the utility of black-and-white filters for optimizing visualization during particular steps in cataract surgery in cases of poor visualization [9].

Herein, we describe an evaluation, in a prospective series, of the efficacy of optimized digital settings for enhancing the visualization of Schlemm’s canal and other iridocorneal structures during surgical gonioscopy.

## 2. Materials and Methods

We prospectively studied patients undergoing iStent inject^®^ implantationat the Guillaume de Varye Hospital (Bourges, France) between June and October 2022. This study conformed to the requirements of the Declaration of Helsinki.

### 2.1. Technique

The same experienced surgeon (O.S.) performed all the operations, using a 3D digital visualization system (NGENUITY^®^, Alcon, Fort Worth, TX, USA). The 3D digital visualization system has an ocular-free design, in which a high-dynamic range video camera connected to a microscope (Lumera 700 Carl Zeiss Meditec, Jena, Germany) replaces the oculars. The surgeon looks directly at a high-definition 3D screen through polarized 3D glasses. An iris diaphragm setting of about 30% was used for the digital video camera in 3D surgery.

All patients underwent surgery under topical anesthesia. In all the cases studied, stent implantation via MIGS was combined with cataract surgery. Ultrasound phacoemulsification was performed with the Constellation (Alcon Surgical, Ft. Worth, TX, USA) microsurgery system equipped with a 0.9 mm 45-degree Kelman mini-flared TurboSonics tip. A 2.2 mm incision was created in the upper part of the cornea, and forceps were used to perform a capsulorhexis of approximately 5.0 mm in diameter. The in-the-bag phaco chop technique was used for nuclear emulsification. After lens implantation, viscoelastic material was injected to deepen the anterior chamber. After rotation of both the patient’s head and the microscope by approximately 30 degrees, the surgeon used his non-dominant hand to place a goniolens iprism^®^ lens (Glaukos) on the corneal surface for visualization of the angle. Intraoperative gonioscopy was then performed to ensure that there was an appropriate open nasal angle for the implantation of the iStent inject^®^ device. Images of the iridocorneal angle were captured with and without the application of the optimized digital settings, and two stents were then implanted in the trabecular meshwork next to Schlemm’s canal under optimized digital visualization, according to the usual practice of the surgeon.

The digital nature of the system allows many adjustments to customize what the surgeon sees during surgery. All image parameters are displayed on the screen and can be modified in real-time, enabling the surgeon to see the effect of these modifications on the enhancement of the tissue of interest (Supplemental Figure S1).

The optimized 3D setting was customized before the start of this study according to the surgeon’s appreciation of the enhancement of visualization for iridocorneal structures. We digitally increased the temperature of the halogen lamp source (to 7000 instead of 3400 Kelvin) relative to the standard color temperature. We also modified the color saturation (to 60% instead of 90%), contrast (to 67.90 instead of 54.90), and saturation of the following filters: the cyan–red filter (to 60% instead of 100%), magenta–green filter (to 70% instead of 100%), and blue–yellow filters (to 60% instead of 100%). With Ngenuity software, only two color channels can be modified simultaneously without interference with the third. We, therefore, first set a filter consisting of the color saturation modifications for magenta and yellow and then applied a cyan color modification to this filter. The other image parameters remained unchanged relative to standard conditions, with brightness set to 47.80, gamma to 1.30, and hue to 2.

### 2.2. Comparison of Surgical Gonioscopy Images

Objective contrast measurements were performed on gonioscopy images recorded with standard and optimized digital settings to demonstrate the digital enhancement of the iridocorneal structures.

We used ImageJ (version 1.53t, (National Institutes of Health (NIH), Bethesda, MD, USA) to draw the regions of interest (ROIs) for the analysis. These ROIs included all the iridocorneal structures: Schwalbe’s line, the non-pigmented and pigmented trabecular meshwork, the scleral spur, and the ciliary body. Variations in pixel intensity, calculated as the differences in the standard deviation (DSD) for pixel intensity, were used to assess contrast.

A subjective analysis was also performed by 2 experienced glaucoma surgeons (O.S. and M.E.) to assess the pigmentation of the trabecular meshwork and the visualization of Schlemm’s canal on a scale of 1 to 3 (1 = worse, 2 = no significant difference, and 3 = better).

### 2.3. Statistical Analysis

The results are expressed as means ± standard deviation for continuous variables and as proportions (%) for discrete variables. The optimized setting and standard-color imaging were compared with paired *t*-tests and non-parametric Wilcoxon’s signed-rank tests for the gonioscopy images.

For the analyses comparing the optimized setting and standard-color conditions, we assessed the agreement between observers with chi-squared tests and by calculating the κ statistic, which estimates the degree of agreement while accounting for that due to chance. We interpreted κ statistics according to the ranges suggested by Landis and Koch: 0 to 0.20, slight agreement; 0.21 to 0.40, fair agreement; 0.41 to 0.60, moderate agreement; 0.61 to 0.80, substantial agreement; and more than 0.80, almost perfect agreement. Values of *p* < 0.05 were considered statistically significant. Statistical analyses were performed with SPSS for Windows version 27.0 (SPSS, Inc., Chicago, IL, USA).

## 3. Results

The series of consecutive case series in this study included 26 eyes of 26 patients undergoing combined iStent implantation via MIGS and cataract surgery. All of the patients had open-angle glaucoma stabilized with one or two drugs. The mean age of the patients was 69.0 ± 5.7 years, and 14 of the 26 eyes were of male patients. Ocular complications were not reported in any of the interventions.

**Figure 1:**
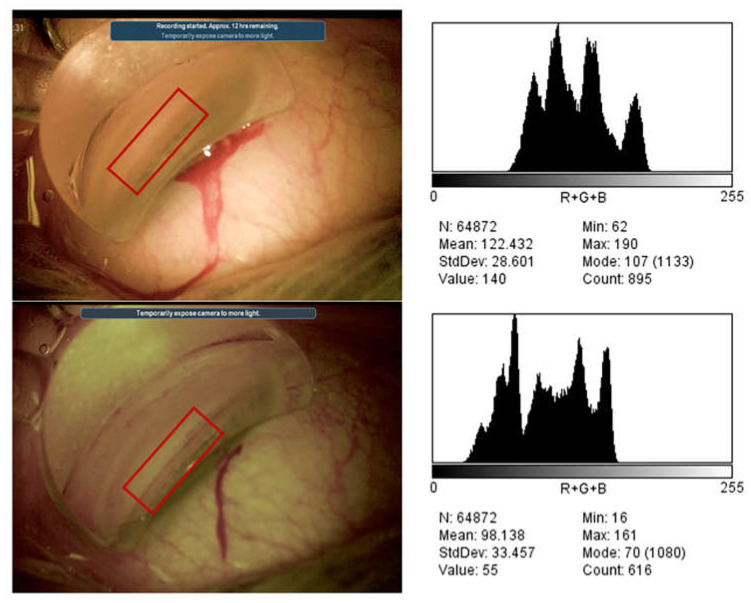
Standard-color images and images obtained with optimized digital settings during surgical gonioscopy and their luminance histograms in pixel values, generated with image J software. The contrast of the region of interest, represented as the difference in the standard deviation (DSD) of pixel intensity values, was 28.601 for standard colors and 33.457 for optimized digital settings.

Gonioscopy image contrast was considered better for optimized filter images than for standard-color images. The mean DSD for pixel intensity was 37.87 (±4.61) for the optimized filter images and 32.37 (±3.51) for the standard-color images (*p* < 0.001) (Figure 1).

**Figure 2:**
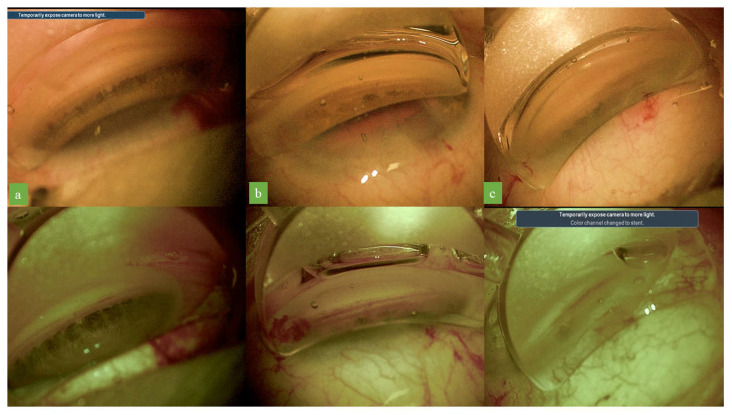
Standard colors gonioscopic images in three cases (**a**–**c**) and corresponding images after optimized digital settings application. Visualization of both the pigmentation of the trabecular meshwork and Schlemm’s canal was enhanced with the optimized digital system.

Very high levels of agreement were observed between the two surgeons (O.S. and E.M.) in the assessment of the visualization quality for trabecular meshwork pigmentation (κ value: 0.83) and for Schlemm’s canal (κ value: 0.90). The surgeons who evaluated the images reported better tissue visualization with the optimized digital settings for both Schlemm’s canal and the pigmentation of the trabecular meshwork in more than 65% of cases (Figure 2). The difference was statistically significant for both trabecular meshwork pigmentation and Schlemm’s canal (*p* < 0.05, chi-square test).

### Tissue Color Modifications

**Figure 3:**
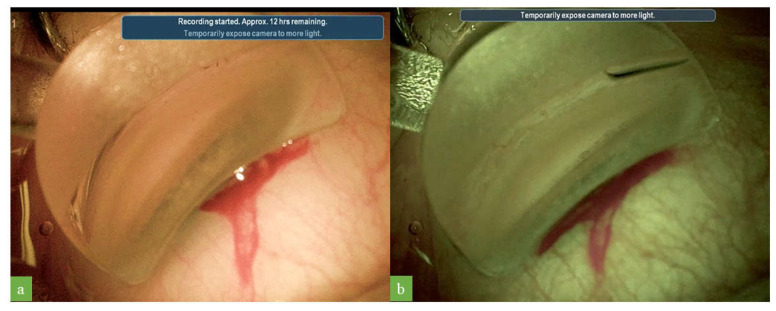
Images obtained during surgical gonioscopy with standard colors (**a**) and with the cyan filter with 60% color saturation (**b**). This filter provided a good level of contrast, enhancing visualization of the pigmentation of the trabecular meshwork and Schlemm’s canal.

**Figure 4:**
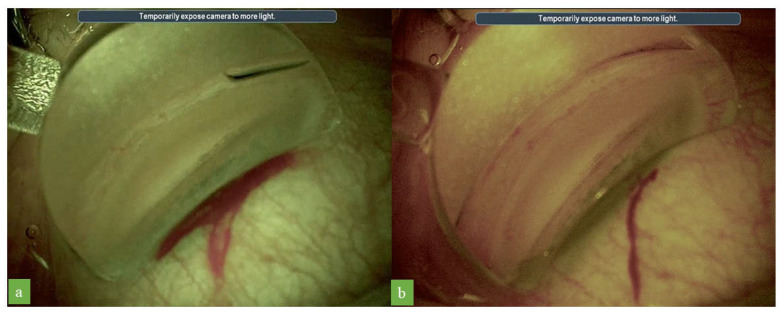
Images obtained during surgical gonioscopy with the optimized settings before (**a**) and after increasing color temperature (**b**). Schlemm’s canal is better visualized, and the addition of warm color tones leads to the canal appearing more red.

The optimized settings differ from standard colors principally in terms of the following three parameters: lower color saturation, greater cyan saturation, and a higher color temperature. The intensity of color fades with 60% color saturation, and the image appears slightly less colorful. This higher cyan saturation provided a good contrast for the pigmentation of the trabecular meshwork and Schlemm’s canal, facilitating the identification of these structures (Figure 3). The increase in color temperature gave the images a warm tone, and Schlemm’s canal, which was grayish in the standard color temperature, appeared reddish (Figure 4). No worsening of the quality of visualization during iStent implantation was observed with the optimized digital filter settings (Supplemental 3D Video).

## 4. Discussion

Over the last few years, MIGS has emerged as an effective tool for glaucoma surgeons. The key to successful MIGS, particularly for the insertion of Schlemm’s canal devices, is good visualization for surgical gonioscopy. The diffuse red hue outlining Schlemm’s canal is often difficult to see, and precise analysis of the trabecular meshwork is more difficult in patients with little or no pigmentation.

Trypan blue dye can be used to stain the trabecular meshwork landmark as a visual guide during implantation via MIGS [10]. However, it induces time-dependent morphological alterations in cultured human trabecular cells following exposure of 60 s or more to the dye [11]. Despite this, there is no evidence of toxicity from shorter exposure, but alternative means for the visualization of iridocorneal structures without the use of dyes would probably be safer and more cost-effective.

Digital technology makes the use of color filters during surgery possible, resulting in better contrast and, thus, a clearer visualization of structures. Several studies have reported improvements in visualization with digital images for both cataract surgery and epiretinal membrane staining during surgery [9,12,13,14].

We, herein, report the utility of optimized digital settings for enhancing the contrast and details of iridocorneal structures based on objective contrast measurements and the subjective analysis of gonioscopic images recorded during MIGS by two glaucoma surgeons.

The three main features of the optimized settings used in our study were greater cyan color saturation, a higher color temperature, and lower color saturation.

Color temperature is a measurement of the color of light emitted by an idealized opaque, non-reflective body at a particular temperature. It is measured in Kelvin. The numerical values, in Kelvin, for color temperature describe the color characteristics of a light source on a spectrum ranging from warm to cool colors [15]. To avoid confusing readers, the color scale is reversed in the Ngenuity system relative to the consensus scale, with the blue color at the bottom of the scale and the orange color at the top [16].

The use of a higher level of cyan saturation provided a good contrast for the visualization of the pigmentation of the trabecular meshwork, facilitating the identification of this structure. Cyan has a low color temperature, whereas the pigmentation of the trabecular meshwork, which tends to be brownish, has a higher color temperature. These colors are almost opposite in terms of their color temperatures, and they tend, therefore, to be complementary (opposite to each other on the color temperature wheel). Complementary colors have the greatest amount of contrast between them [17]. Indeed, in our study, we observed an enhancement of the visualization of the pigmentation in the trabecular meshwork with the cyan color filter.

**Figure 5:**
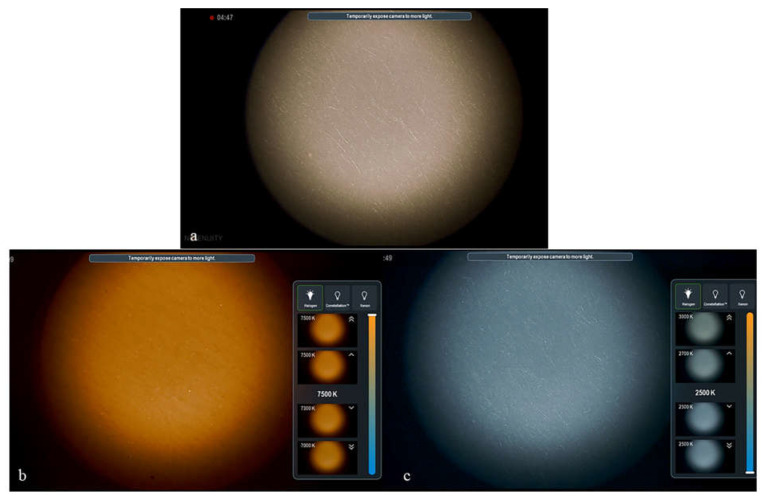
Effect of changing color temperature with the Ngenuity system on the appearance of a sheet of white paper (**a**). The paper takes on a warm, orange tint when the color temperature is increased to 7500 K (**b**) and a blue cool tint when the color temperature is decreased to 2500 K (**c**).

The use of Ngenuity digital visualization technology made it possible to also increase the color temperature of the images. At neutral lighting color temperatures, white objects appear white in images. However, if we increase the color temperature of the illumination, white objects appear orange in the image. Conversely, if we decrease the color temperature, white objects appear with a cooler blue tone (Figure 5).

Increasing the color temperature adds a warm tone to an image, making the colors in the image appear warmer. As demonstrated in this study, by increasing temperature colors in the images, Schlemm’s canal, which is barely visible with standard colors, becomes reddish in appearance and, therefore, easier to identify. Blood flowing within Schlemm’s canal is sometimes visible as a red line with standard colors. However, the use of a higher color temperature for imaging renders the color of the blood warmer and more reddish, making it easier to detect. The enhanced visualization of Schlemm’s canal may increase the accuracy and safety of stent implantation via MIGS, which may potentially improve postoperative results.

Color saturation was slightly decreased digitally to improve contrast. It has been reported that decreases in color saturation can improve the delimitation of tissue contours in visualizations, facilitating internal limiting membrane peeling and enhancing visualization during cataract surgery [9,18].

The magenta filter was used to highlight colors close to magenta, thereby enhancing the visualization of blood within Schlemm’s canal, and to compensate for the decrease in the color intensity of Schlemm’s canal due to the 60% decrease in color saturation.

The digital color system of the Ngenuity system can also modify image quality by changing other parameters, such as contrast and hue, and through the use of the yellow filter, as used here. However, we found that altering these parameters enhanced iridocorneal visualization less strongly and less consistently than the main parameters cited before (cyan filter, color temperature, and color saturation). Nevertheless, all parameters can be modified to suit the surgeon’s personal preferences in each individual case of surgical gonioscopy, depending on the pigmentation of the trabecular meshwork and Schlemm’s canal visualization.

In conclusion, we described herein an optimized setting for use with a digital 3D visualization system to enhance the visualization of iridocorneal structures during gonioscopy. We found that the use of cyan filters and the modifications of saturation and temperature color profiles improved the visualization of Schlemm’s canal and the trabecular meshwork. This approach could be of potential value for increasing the accuracy of MIGS device implantation.

## Data Availability

The data that support the findings of this study are available from the corresponding author, S.O. upon request.

## Supplementary Materials

The following supporting information can be downloaded at https://www.mdpi.com/article/10.3390/jcm12082794/s1: Supplemental 3D Video: Video of several cases, highlighting the digital enhancement of visualization of the iridocorneal angle structures in stent implantation surgery.
