# Supplementary figures and images for: Use of Digital Methods to Optimize Visualization during Surgical Gonioscopy
Source: J Clin Med. 2023 Apr 10;12(8):2794. doi: 10.3390/jcm12082794 (PMC10146903; doi:10.3390/jcm12082794)

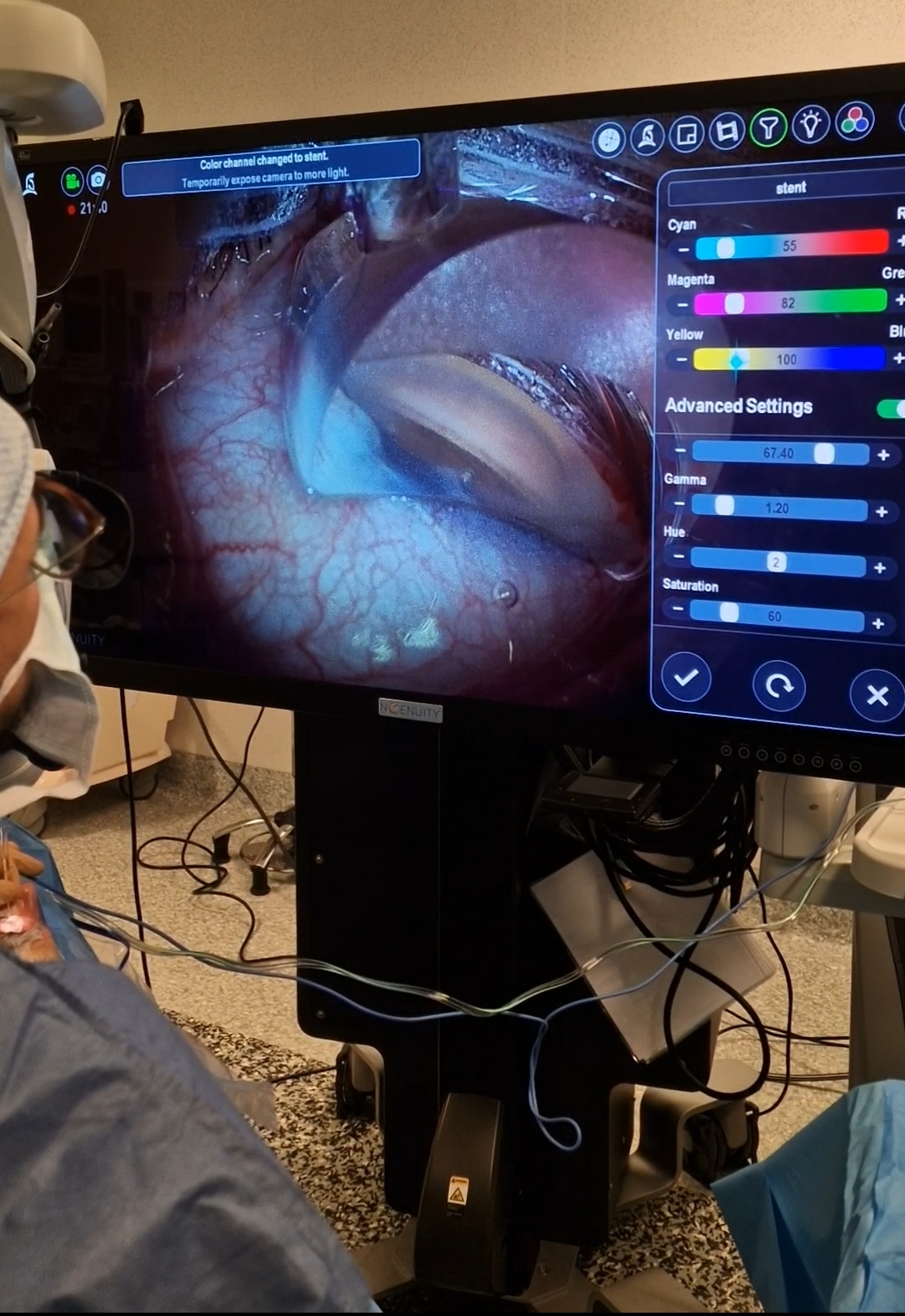

Supplement: Supplementary file 1 [file jcm-12-02794-s001.zip › Supplemental Figure S1.tiff]
